# Role of the Domestic Chicken (Gallus gallus)in the Epidemiology of Urban Visceral Leishmaniasis in Brazil

**DOI:** 10.3201/eid0812.010485

**Published:** 2002-12

**Authors:** Bruce Alexander, Renata Lopes de Carvalho, Hamish McCallum, Marcos Horácio Pereira

**Affiliations:** * Centro de Pesquisas René Rachou, Belo Horizonte, Brazil; † Pontifícia Universidade Católica de Minas Gerais, Belo Horizonte, Brazil; ‡ University of Queensland, Brisbane, Australia; § Universidade Federal de Minas Gerais, Belo Horizonte, Brazil

**Keywords:** leishmaniasis, Brazil, urban, chickens, Phlebotominae

## Abstract

Zoonotic visceral leishmaniasis (ZVL) is a serious public health problem in several Brazilian cities. Although the proximity of chicken houses is often cited as a risk factor in studies of urban ZVL, the role chickens play in the epidemiology of the disease has not been defined. Chickens attract both male and female sand flies (Lutzomyia longipalpis), but are unable to sustain Leishmania infections, and their presence may exert a zooprophylactic effect. We discuss environmental, physiologic, socioeconomic, and cultural factors related to chicken raising that could influence Le. infantum transmission in Brazilian cities and evaluate whether this practice significantly affects the risk of acquiring ZVL.

During the last 20 years, zoonotic visceral leishmaniasis (ZVL) due to Leishmania (Leishmania) infantum has become a serious public health problem in several Brazilian cities (1). The pathogen is transmitted by the bite of the phlebotomine sand fly Lutzomyia longipalpis (Lutz & Neiva 1912), and although humans can be infected, they are believed to be “dead-end” hosts; domestic dogs are the main reservoirs for the parasite. The spread and increasing prevalence of ZVL in urban areas are linked to human migrations, involving the transportation of infected dogs from ZVL-endemic regions to impoverished urban areas where Lu. longipalpis already exists. Although generally located on the margins of large Brazilian cities, these shanty towns (favelas) in Belo Horizonte (population 2.3 million) are dispersed throughout the urban zone, often adjacent to wealthy neighborhoods. Many of the inhabitants raise chickens, pigs, and other livestock in their yards, and because of the general climate of insecurity, keep dogs, which act as amplification hosts for Le. infantum (2). Thus all the factors for parasite transmission may be concentrated within a relatively small area.

The proximity of hen houses is acknowledged as a possible environmental risk factor in studies of urban ZVL (3,4), but the role chickens play in Le. infantum transmission has not been completely explained. A study in the Brazilian state of Bahia found that dwellings of persons with ZVL were 4.21 times as likely to have chicken houses in the yard as those whose occupants were unaffected (5), but other studies have failed to demonstrate a significant correlation. Although the attraction of chickens for Lu. longipalpis is indisputable, chickens, like other birds, are unable to sustain infections with Leishmania, and the nature of the relationship between chicken raising and ZVL is complex. In this article, we consider factors related to raising chickens that might affect transmission of Le. infantum in Brazilian cities and discuss whether raising chickens in urban areas could affect the risk of human acquisition of ZVL.

## Importance of Sand Fly Attraction to Chickens in Le. infantum Transmission

Widely differing observations regarding the degree to which Lu. longipalpis bites humans in different habitats, as well as the fact that female sand flies from nonanthropophilic populations can be induced to feed on humans in the laboratory indicate that this species has no strong innate host preference. Although sand flies in Brazil are known by a number of common names, including canagalinha, mosquito de palha, and asa branca, the absence of such a term to distinguish Lu. longipalpis from other biting flies in urban ZVL foci suggests that this fly does not constitute a substantial biting nuisance for the inhabitants.

Sand fly reproduction depends on the availability of blood meal sources such as domestic animals and synanthropic species that raid chicken houses and are potential reservoirs of Le. infantum, such as the fox (Cerdocyon thous), opossum (Didelphis albiventris), and black rat (Rattus rattus) (2,6). Although involved in sylvatic transmission of Leishmania, the fox is less likely to be found in urban areas than the other two species. Host loyalty involving subpopulations of vectors would have a marked effect on Leishmania transmission (7). The relative attractiveness of chickens compared with other hosts at a particular site can be calculated from “forage ratios” (8), in which the percentage of sand flies feeding on the birds is divided by their relative numerical importance: values significantly >1.0, indicating selective preferences. A study in rural Colombia (9) demonstrated that Lu. longipalpis clearly preferred pigs and cows over chickens (values were < 0.75). However, results of such studies may not be reproducible in other situations, where wind direction and the relative proximity of different hosts to host-seeking sand flies affect attraction. Comparisons of the attractiveness of different host species should also take into account differences in biomass, heat loss (a function of the surface area/volume ratio), and CO2 production (10). Chickens produce 19–26 m3/kg body weight of C02 per minute (11); comparable figures can be estimated as 13–17 for dogs and 8–11 for humans, when specific metabolic rate scales (in homeotherms) are used as mass-0.25 (12). Field experiments on Marajo Island, Brazil, showed that one boy attracted significantly more female Lu. longipalpis than one dog or one chicken and slightly fewer sand flies than six chickens (13). Assuming that the children participating in this study each weighed about 40 kg and chickens 2 kg, then the amounts of C02 produced by one boy would be approximately 400 m3/min, equivalent to that of about eight chickens. However, host odor is probably the most important stimulus for orientation of blood-feeding insects in open (i.e., nonforest) situations (14).

Flights of several hundred meters have been recorded for Lu. Longipalpis, and infected sand flies attracted to an area by chicken houses may be diverted en route or displaced to other hosts. This diversion would explain the presence of (usually canine) ZVL in wealthy districts adjacent to poorer neighborhoods, a pattern seen in many Brazilian cities. Male sand flies marked with fluorescent powders traveled distances of up to 430 m between chicken houses in Montes Claros, Brazil (Kirby M. American visceral leishmaniasis—the importance of the domestic chicken Gallus gallus to the urban distribution of the sandfly vector Lutzomyia longipalpis [Diptera: Psychodidae] [M.Sc. thesis]. London: London School of Hygiene and Tropical Medicine; 2000), so such flights are clearly not limited to females in search of blood meals.

## Zooprophylaxis and Factors Precluding Chickens as Hosts of Leishmania

Chickens have several physiologic characteristics that preclude them from sustaining Leishmania infections, including their body temperature of 41.0°C (15). Enzymatic processes in the sand fly function differently when triggered by different types of blood meal, and blood from certain sources may be lethal to Leishmania (16). Turkey blood meals significantly reduced Le. tropica infections in the Old World sand fly Phlebotomus papatasi, even when insects were infected after digestion of the blood meal, perhaps due to DNAase activity triggered by the presence of nucleated erythrocytes. A few drops of turkey blood rapidly killed Le. tropica promastigotes in culture, although this in vitro effect could not be the same as that in the sand fly gut and may be complement-related (17,18). Thus, not only is Leishmania infection unable to develop in birds, but also existing infections might be eliminated in sand flies taking a second blood meal from chickens.

For a single host species, the basic reproductive rate R0 of a vector-transmitted pathogen is given by the following equation (19),
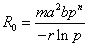
where m is the number of vectors per host, a is the daily biting rate of each individual vector on the host species, b is the fraction of infected vectors that actually generate infection when biting a susceptible host, p is the daily survival rate of the vectors, n is the latent period of infection in the vectors, and r is the daily recovery rate of the hosts. When host species are numerous, R0 can be derived in general from the dominant eigenvalue of a modified “who acquires infection from whom” matrix (20). In the special case when one host (such as a chicken) is a dead end, its presence does not influence the mathematical form of eigenvalue: rather, the question is what influence this host has on a and m. The proximity of chickens to humans may potentially increase m by attracting more sand flies into the local area, or even by maintaining a higher sand fly population, through provision of additional resources. Other dead-end hosts will decrease a on humans (a zooprophylactic effect) because a given sand fly will be able to obtain its nutrition from an alternative source. Which effect dominates depends on the relative strength of these competing effects; note that a enters into R0 as a square, compared with m, which has a linear effect. A further complication is that, if the presence of chicken houses in some areas has the effect of aggregating an existing sand fly population, an overall increase in R0 will result (21)

## Chickens as Blood Meal Sources for Maintenance of Sand Fly Populations

Although chickens cannot act as Le. infantum reservoirs, they may be important in maintaining vector populations and attracting mammalian reservoirs to the vicinity. Feeding success of sand flies can be measured by using the equation Gi = Qi/Nmj (22), where Gi is the mean gain in resources (e.g., blood meal size) on host i, Q is an estimate of patch quality (in this case, number of chickens) and Nmi, the biting rate. Nutritional quality of blood (about 90% protein by dry weight) varies between host species and Gi may also be revealed by reduced rates of development, longevity, and digestion, as well as skewed by sex ratios (14). Laboratory studies of fecundity of insects fed on blood from different hosts often fail to take into account natural factors such as host defense mechanisms (both behavioral and physiologic), activity patterns, and intra- or interspecific competition at feeding sites. Although no comparative studies of fecundity involving sand flies fed on birds exist, the mosquito Culex pipiens produced twice as many eggs per mg of blood when fed on canaries as when fed on humans (23). The results of this study notwithstanding, avian blood should be less nutritious than that of mammals for several reasons. Chicken erythrocytes are nucleate and have a DNA content 31 times that found in humans. They also have a lower hemoglobin content than mammalian red cells and a hematocrit value half that of mammals. These values mean that sand flies feeding on chickens would have to ingest twice as much blood as those on mammals to obtain a meal containing the same quantity of erythrocytes. Unlike mosquitoes, sand flies do not expel any of the blood meal while feeding and cannot continue to engorge when replete (24). Even if plasma rather than erythrocytes were the essential component for ovarian development (25), total plasma protein levels in chickens are considerably lower than in dogs and pigs. In addition, catabolism of nucleic acids from chicken erythrocytes would presumably involve greater bio-energetic costs due to increased production and active transport of uric acid, the end product of nitrogen metabolism in insects (26).

## Factors Favoring or Limiting the Feeding of Lu. longipalpis on Chicken Blood

Unlike most mammal species, chickens are inactive at night and present large areas of exposed skin on which sand flies can feed. The comb and wattles are richly supplied with capillaries but the epidermis is much thinner (~0.02 mm) on feathered areas of the body (27) and could thus be pierced more easily by the proboscis of a sand fly. Sand fly mouthparts are too short to probe deeper than the superficial loops of the host’s capillaries, and the insects ingest blood from pools that form after laceration of the ends of the vessels (28). This mode of feeding exposes the female sand fly to a battery of hemostatic and inflammatory reactions, and saliva of Lu. longipalpis contains substances able to counteract these, including anticoagulants, apyrase to inhibit platelet aggregation and a potent vasodilator (29,30). The erythrocytes are relatively soft and easily ruptured, while the thrombocytes, which are analogous to platelets in mammals, are less efficient in reducing blood loss in birds (31). These characteristics could facilitate blood feeding by sand flies, as has been observed in triatomines (32). Reductions in blood flow rate due to colonization of the pharynx and cibarium by Leishmania (33) could also make feeding on chickens preferable for infected sand flies, further favoring zooprophylaxis of ZVL.

## Chicken Houses as Foci of Reproductive Behavior for Lu. longipalpis

Male blood-sucking flies that are irregularly or widely dispersed in a habitat may gain a mating advantage by staying with the host and waiting for females to arrive (34). Male Lu. longipalpis encountered on a host at a particular moment usually far outnumber females, and courtship behavior involves mating aggregations or “leks” where males compete by producing sex pheromones. The effective range of the compounds involved (35) is a function of their volatility; less volatile molecules are active over shorter distances but produce a more coherent message (J.G.C. Hamilton, pers. comm.).

Preliminary trials of a pheromone-baited trap for Lu. longipalpis obtained better results when extracts were heated (36), and host temperature might be important in disseminating these compounds. Chickens’ higher body temperature could thus favor them over mammals as lekking sites for male Lu. longipalpis. In view of the short effective range of male pheromones (~2 m), pheromones are unlikely to be involved in attracting sand flies to chicken houses rather than host-produced stimuli such as odor and CO2 that extend for further distances.

Newly emerged Lu. longipalpis adults and larvae of several Old World species have been collected in animal shelters (37). However, attempts to recover larvae from chicken houses have been unsuccessful (38), perhaps because the nitrogen-rich feces of chickens are unpalatable to them. Lu. longipalpis adults may rest in chicken houses after taking blood but breed in nearby, less accessible microhabitats such as rodent burrows, where temperature, relative humidity, and light levels are more constant. Oviposition of laboratory-raised Lu. longipalpis involves a thigmotropic response (39), suggesting that in the wild females lay eggs in confined spaces such as crevices rather than on exposed surfaces. No evidence is available on predatory behavior by chickens toward sand flies or their natural enemies (which are largely unknown).

## Interventions Focused on Chicken Houses

Chicken houses are sprayed with residual insecticides as part of the current ZVL control strategy in Brazil (40), but this spraying is constrained by costs of materials and availability of trained personnel. An alternative would be to modify the environmental factors favoring contact between vectors, reservoirs, and susceptible humans, such as proximity to chicken houses. A similar approach has been suggested for controlling dengue (41), which currently afflicts the same segment of the Brazilian population as ZVL.

With regard to conventional control programs, the relative merits of insecticidal spraying of human dwellings, chicken houses, or both, need to be considered. DDT spraying of houses in the Brazilian Amazon region failed to reduce the incidence of cutaneous leishmaniasis, perhaps because most of the vectors (Lu. intermedia) rested in chicken coops, which were left untreated (42). Presumably, the numbers of sand flies that did not feed on chickens were sufficiently large to balance any zooprophylactic effect, and chicken coops may only have been used as resting sites. Spraying houses alone would be an effective strategy only if all female sand flies in the vicinity could be diverted to feeding on chickens. However, spraying chicken houses alone would probably be ineffective because the odor and CO2 produced by the birds would still attract sand flies to the vicinity, and the sand flies risk encountering infected or susceptible mammals (including humans) en route and when they rest afterwards in untreated microhabitats.

## Socioeconomic Importance of Chicken Rearing

Alexander et al. (unpubl. data) found that up to 27.0% of residents of poor neighborhoods in the city of Montes Claros kept chickens for the following reasons: to produce eggs (50.0%) or meat (34.5%) for occasional personal consumption; as a hobby (23.6%); for cock-fighting (3.6%); to keep yards free of trash (9.1%); or to control scorpions (Tityus serrulatus) (7.3%). Nevertheless, 84.6% of the people interviewed said they would stop raising chickens if it was proved that keeping chickens increased the risk of acquiring ZVL.

No information is available on the contribution of poultry products to children’s nutrition in urban foci of Le. infantum transmission. In any case, ZVL is more likely to develop in children with moderate or severe malnutrition than in healthy children (43), and infant malnutrition is common in Brazilian cities (values of 25.9% and 19.7% are recorded for the state capitals of São Paulo and Curitiba, respectively) (44). If families derive a large proportion of their daily protein intake from chickens or eggs, prohibition of raising chickens might therefore affect the prevalence of clinical manifestations of ZVL in infected children. Current legislation that bans livestock within the limits of Brazilian cities often does not specifically prohibit poultry raising.

## Conclusions

 Lane (42) discussed a number of the points mentioned in this article, noting that the relationship between chicken houses and sand flies also extended to the Old World Leishmania vectors Phlebotomus argentipes, P. langeroni, P. ariasi, and P. papatasi. In Brazil several other Lutzomyia species also feed on chickens or at least rest in chicken houses, including the Le. braziliensis vectors Lu. intermedia and Lu. whitmani, so that the shelters clearly offer important man-made refuges for sand flies in urban environments. Nonetheless, the results of epidemiologic studies that attempt to incriminate chickens as a risk factor for urban ZVL are conflicting. Since the disease is potentially fatal, as is Chagas disease, zooprophylaxis as a means of control cannot be tested experimentally for ethical reasons.

 The relationship between chicken raising and Le. infantum transmission by sand flies is summarized in the Table. Modeling the risk of Leishmania transmission by sand flies associated with chickens would require collecting field or laboratory data on all the factors discussed above, but current knowledge can be summarized as follows. Chicken houses attract both blood-seeking females and males seeking mates, but do not appear to act as breeding sites. They also attract potential reservoirs of Leishmania and are protected by dogs, themselves amplification hosts of the parasite. Nevertheless, chickens are refractory to Leishmania infection and, in certain situations, act as zooprophylactic agents. Although chicken blood may be less nutritious than that of mammals, influencing egg productivity and thus population levels of sand flies, this disadvantage would be compensated to some extent by the greater facility with which Lu. longipalpis is able to feed on birds. Prohibiting chicken rearing in Brazilian cities would remove a potential source of food and income for the inhabitants of low-income neighborhoods. In fact, some health authorities currently advise householders to keep only two chickens to control scorpions, although no published data support this recommendation. A recent study modeled Trypanosoma cruzi transmission among populations of humans, dogs, and chickens in three Argentinian villages (45), a situation that may be considered analogous to that of urban ZVL foci in Brazil. Prevalence of infection decreased slowly as the fraction of triatomine bugs feeding from chickens increased, indicating a slight zooprophylactic effect. In addition, as the relative density of the bugs increased, the proportion that fed on humans rather than chickens decreased.

**Table:** Positive and negative factors associated with chicken raising that may affect the transmission of Leishmania infantum by Lutzomyia longipalpis in urban foci of zoonotic visceral leishmaniasis, Brazil

Factors affecting risk of transmission of Le. infantum to humans (all+)	Factors affecting risk of infection of Lu. longipalpis by Le. infantum (all-)	Factors affecting maintenance of sand fly populations (+/-)		
		Facilitation of blood feeding (+)	Nutrition (-)	Bringing sexes together (+)
Chicken rearing seen by local people as providing several benefits not directly related to Leishmania transmission, e.g., scorpion control, source of food and income and keeping yards free of trash Odor and CO2 emitted by chickens attract infected sand flies to the vicinity of human dwellings Presence of chickens attracts potential reservoirs of Le. infantum to the vicinity of human dwellings where sand flies also present Dogs kept to guard chicken houses from thieves and predators are themselves potential reservoirs of Le. Infantum Chicken houses may act as resting sites for engorged sand flies No evidence that chicken houses act as sand fly breeding sites although associated rodent burrows might be exploited	Complement levels in blood fatal to Leishmania? Temperature of chicken blood too high (41°C) to permit growth of Leishmania Greater facility with which sand flies can feed on chickens would favor biting by infected sand flies whose capacity to ingest blood is compromised by blockage of the pharynx? Nucleated erythrocytes in blood meal stimulate DNAase activity fatal to Leishmania within sand fly gut?	Chicken RBCs soft and easily ruptured Chickens easier to feed on by sand flies that have pharynx partially blocked by Leishmania? Chicken skin thinner than that of mammals (0.02 mm), esp. on feathered areas of body Thrombocytes less efficient than mammalian platelets in preventing blood loss	Protein content of chicken plasma considerably lower than that of mammals Nucleated blood cells have 31 times DNA content of human erythrocytes; elimination associated w/ problems of water balance? Hematocrit value of chicken blood about 50% that of mammals; sand flies cannot concentrate blood meal during engorgement	CO2 and odor attractive to both males and females High chicken body temperature might favor dissemination of pheromone Passivity of roosting chickens lets male sand flies display relatively undisturbed

aRBCs, red blood cells; esp., especially.

Urban ZVL is an increasingly grave public health problem in Brazil that imposes an additional strain on local health authorities and is unlikely to be resolved by current strategies. Chickens are the most common type of livestock raised in low-income neighborhoods. Understanding the role of chicken raising in the Le. infantum transmission cycle could lead to inexpensive and sustainable preventive measures, perhaps involving the acquiescence of local people in the removal or focal treatment of chicken houses. The role played by chickens in the epidemiology of urban ZVL clearly involves some type of balance between zooprophylaxis, maintenance of sand fly populations, and attraction of reservoir hosts of Le. infantum. This balance may vary in different situations but could be further clarified by the following activities: 1) field observations to determine the relative importance of chickens and other hosts as blood meal sources and lekking sites; 2) laboratory studies of comparative egg productivity of sand flies fed on chickens and other hosts; and 3) socioeconomic surveys on the importance of chickens to communities affected by ZVL in terms of income and nutrition, as well as communities’ willingness to participate in preventative measures e.g., removal of chicken houses.
